# Supplementary material for: Durability of mRNA-1273-induced antibodies against SARS-CoV-2 variants
Source: bioRxiv. 2021 May 16:2021.05.13.444010. Preprint. [Version 1] doi: 10.1101/2021.05.13.444010 (PMC8142657; doi:10.1101/2021.05.13.444010)
Supplement: 1 [file NIHPP2021.05.13.444010V1-supplement-1.pdf]

bioRxiv preprint doi: https://doi.org/10.1101/2021.05.13.444010; this version posted May 16, 2021. The copyright holder for this preprint (which was not certified by peer review) is the author/funder. This article is a US Government work. It is not subject to copyright under 17 USC 105 and is also made available for use under a CC0 license.

Supplementary Text

mRNA-1273 Study Group

The following study group members were all closely involved with the design, implementation, and oversight of the mRNA-1273 clinical trial.

Division of Microbiology and Infectious Diseases, National Institute of Allergy and Infectious Diseases, National Institutes of Health, Bethesda, MD. Jae Arega, M.S., John H. Beigel, M.D., Wendy Buchanan, M.S., B.S.N., Mohammed Elsafy, M.D., Binh Hoang, Pharm.D., Rebecca Lampley, M.Sc., Aparna Kolhekar, Ph.D., Hyung Koo, B.S.N., Catherine Luke, Ph.D., Mamodikoe Makhene, M.D., M.P.H., Seema Nayak, M.D., Rhonda Pikaart-Tautges, B.S., Paul C. Roberts, Ph.D., Janie Russell, B.S., Elisa Sindall, B.S.N.

The Emmes Company, LLC, Rockville, MD. Jim Albert, M.S., Pratap Kunwar, M.S., Mat Makowski, Ph.D.

Emory University School of Medicine, Atlanta, GA. Evan J. Anderson, M.D., Amer Bechnak, M.D., Mary Bower, R.N., Andres F. Camacho-Gonzalez, M.D., M.Sc., Matthew Collins, M.D., Ph.D., Ana Drobeniuc, M.P.H., Venkata Viswanadh Edara, Ph.D., Srilatha Edupuganti, M.D., M.P.H, Katharine Floyd, Theda Gibson, M.S., Cassie M. Grimsley Ackerley, M.D., Brandi Johnson, Satoshi Kamidani, M.D., Carol Kao, M.D.; Colleen Kelley, M.D., M.P.H., Lilin Lai, M.D., Hollie Macenczak, R.N., Michele Paine McCullough, M.P.H., Etza Peters, R.N., Varun K. Phadke, M.D., Paulina A. Rebolledo, M.D. M.Sc., Christina A. Rostad, M.D., Nadine Rouphael, M.D., Erin Scherer Ph.D., D.Phil., Amy Sherman, M.D., Kathy Stephens, R.N., Mehul S. Suthar, Ph.D., Mehgan Teherani, M.D., M.S., Jessica Traenkner, P.A., Juton Winston, Inci Yildirim, M.D., Ph.D.

Kaiser Permanente Washington Health Research Institute, Seattle, WA. Lee Barr, R.N., Joyce Benoit, R.N., Heather Beseler, M.B.A., Rachael Burganowski, M.S., Barbara Carste, M.P.H., Joe Choe, B.S., John Dunn, M.D., M.P.H., Maya Dunstan, M.S., R.N., Roxanne Erolin, M.P.H., Jana ffitch, L.P.N., Colin Fields, M.D., Lisa A. Jackson, M.D., Erika Kiniry, M.P.H., De Vona Lang, L.M.P., Susan Lasicka, R.Ph., Stella Lee, B.A., Matthew Nguyen, M.P.H., Jennifer Nielsen, M.N., A.R.N.P., Hallie Phillips, M.ed., Stephanie Pimienta, B.S., David Skatula, R.Ph., Janice Suyehira, M.D., Karen Wilkinson, M.N., A.R.N.P., Michael Witte, Pharm.D.

Moderna, Inc., Cambridge, MA. Hamilton Bennett, M.Sc., Nedim Emil Altaras, Ph.D., Andrea Carfi, Ph.D., Marjorie Hurley, Pharm.D., Brett Leav, M.D., Rolando Pajon, Ph.D., Wellington Sun, M.D., Tal Zaks, M.D., Ph.D.

Seattle Children's Research Institute, Seattle, WA. Rhea N. Coler, M.Sc., Ph.D., Sasha E. Larsen, Ph.D.

University of Maryland School of Medicine, Baltimore, MD. Kathleen M. Neuzil, M.D.

bioRxiv preprint doi: https://doi.org/10.1101/2021.05.13.444010; this version posted May 16, 2021. The copyright holder for this preprint (which was not certified by peer review) is the author/funder. This article is a US Government work. It is not subject to copyright under 17 USC 105 and is also made available for use under a CC0 license.

University of North Carolina, Durham, NC. Lisa C. Lindesmith, M.S., David R. Martinez, Ph.D., Jennifer Munt, B.S., Michael Mallory, M.P.H., Caitlin Edwards, B.S., Ralph S. Baric, Ph.D.

Vaccine Research Center, National Institute of Allergy and Infectious Diseases, National Institutes of Health, Bethesda, M.D. Nina M. Berkowitz, M.P.H., Kevin Carlton, M.S., Kizzmekia S. Corbett, Ph.D., Pamela Costner, R.N., B.S.N., Nicole A. Doria-Rose, Ph.D., Britta Flach, Ph.D., Martin Gaudinski, M.D., Ingelise Gordon, R.N., Barney S. Graham, M.D., LaSonji Holman, F.N.P., Julie E. Ledgerwood, D.O., Kwanyee Leung, Ph.D., Bob C. Lin, B.S., Mark K. Louder, John R. Mascola, M.D., Adrian B. McDermott, Ph.D., Kaitlyn M. Morabito, Ph.D., Laura Novik, R.N., M.A., Sarah O'Connell, M.S., Sijy O'Dell, M.S., Marcelino Padilla, B.S., Amarendra Pegu, Ph.D, Stephen D. Schmidt, B.S., Phillip A. Swanson II, Ph.D., Chloe A. Talana, B.S., Lingshu Wang, Ph.D., Alicia T. Widge, M.D., M.S., Eun Sung Yang M.S., Yi Zhang B.S.

Vanderbilt University Medical Center, Nashville, TN. James D. Chappell, M.D., Ph.D., Mark R. Denison, M.D., Tia Hughes, M.S., Xiaotao Lu, M.S., Andrea J. Pruijssers, Ph.D., Laura J. Stevens, M.S.

Fred Hutchinson Cancer Research Center, Seattle WA. Christine M. Posavad, Ph.D

University of Washington, Seattle, WA. Michael Gale, Jr., Ph.D.

University of Texas Medical Branch, Galveston, TX. Vineet Menachery, Ph.D., Pei-Yong Shi, Ph.D.

bioRxiv preprint doi: https://doi.org/10.1101/2021.05.13.444010; this version posted May 16, 2021. The copyright holder for this preprint (which
was not certified by peer review) is the author/funder. This article is a US Government work. It is not subject to copyright under 17 USC 105
and is also made available for use under a CC0 license.

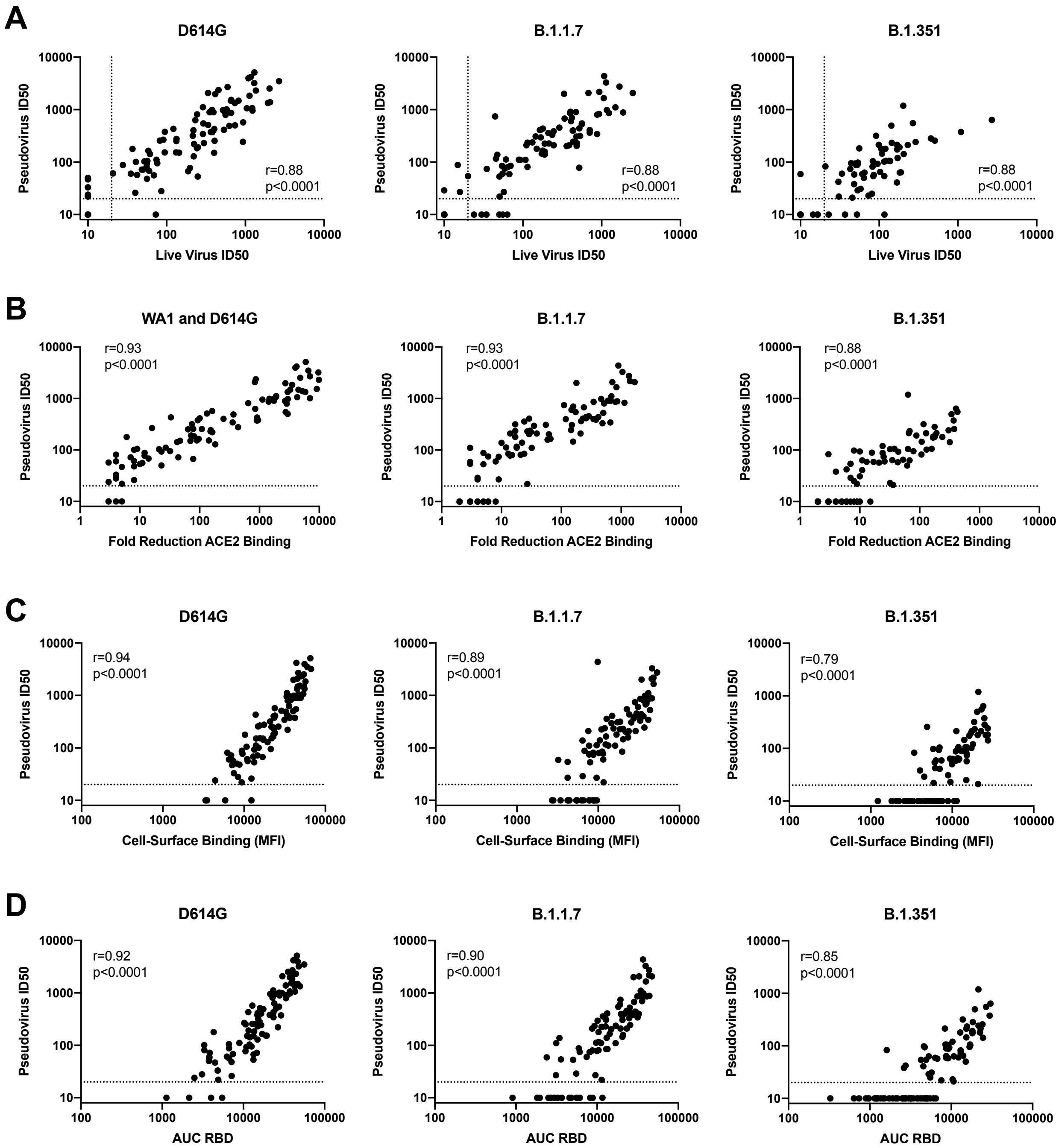

Fig. S1.

Functional and binding assays correlate well with each other. Each graph shows n=96 serum samples. r values: Spearman's rho. Graphs show pseudovirus neutralization compared to: **A,** live-virus FRNT ID50, **B**. fold reduction in ACE2 binding, **C**. cell-surface binding median fluorescence intensity (MFI), **D.** binding to RBD in MSD-ECLIA assay, expressed as area under the curve (AUC). Left: WA1 or D614G; middle, B.1.1.7; right, B.1.351.

bioRxiv preprint doi: https://doi.org/10.1101/2021.05.13.444010; this version posted May 16, 2021. The copyright holder for this preprint (which was not certified by peer review) is the author/funder. This article is a US Government work. It is not subject to copyright under 17 USC 105 and is also made available for use under a CC0 license.

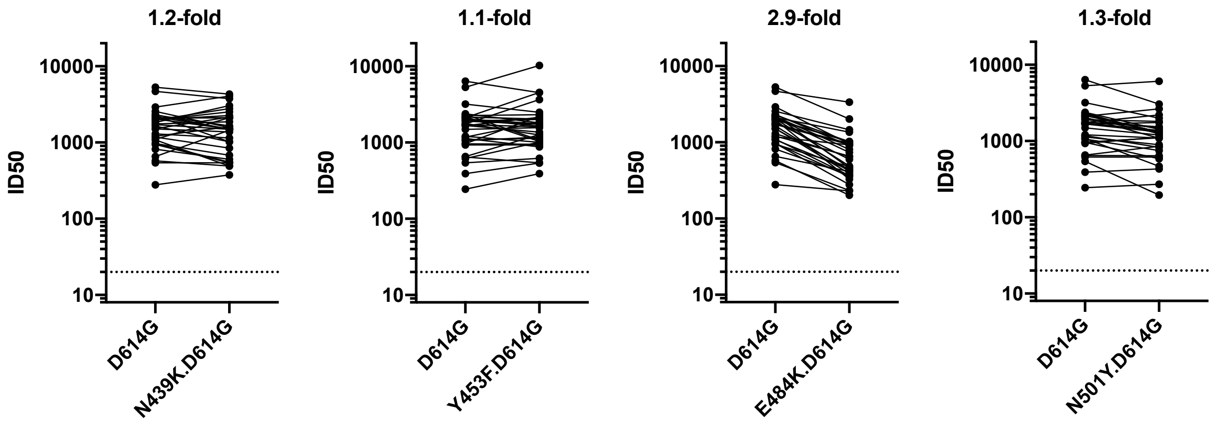

Fig. S2.

Point mutations cause modest decreases in neutralizing activity.

Day 43 Sera were assessed in lentivirus-based pseudovirus neutralization assay. 33 sera were tested, inclusive of the 24 used in other figures plus additional samples as described in (20). Pseudoviruses were: D614G, D614G.N439K, D614G.Y453F, D614G.E484K, and D614G.N501Y. For each pair of viruses, the fold-differenec is the geometric mean of the ratio of ID50s for each serum.

bioRxiv preprint doi: https://doi.org/10.1101/2021.05.13.444010; this version posted May 16, 2021. The copyright holder for this preprint (which was not certified by peer review) is the author/funder. This article is a US Government work. It is not subject to copyright under 17 USC 105 and is also made available for use under a CC0 license.

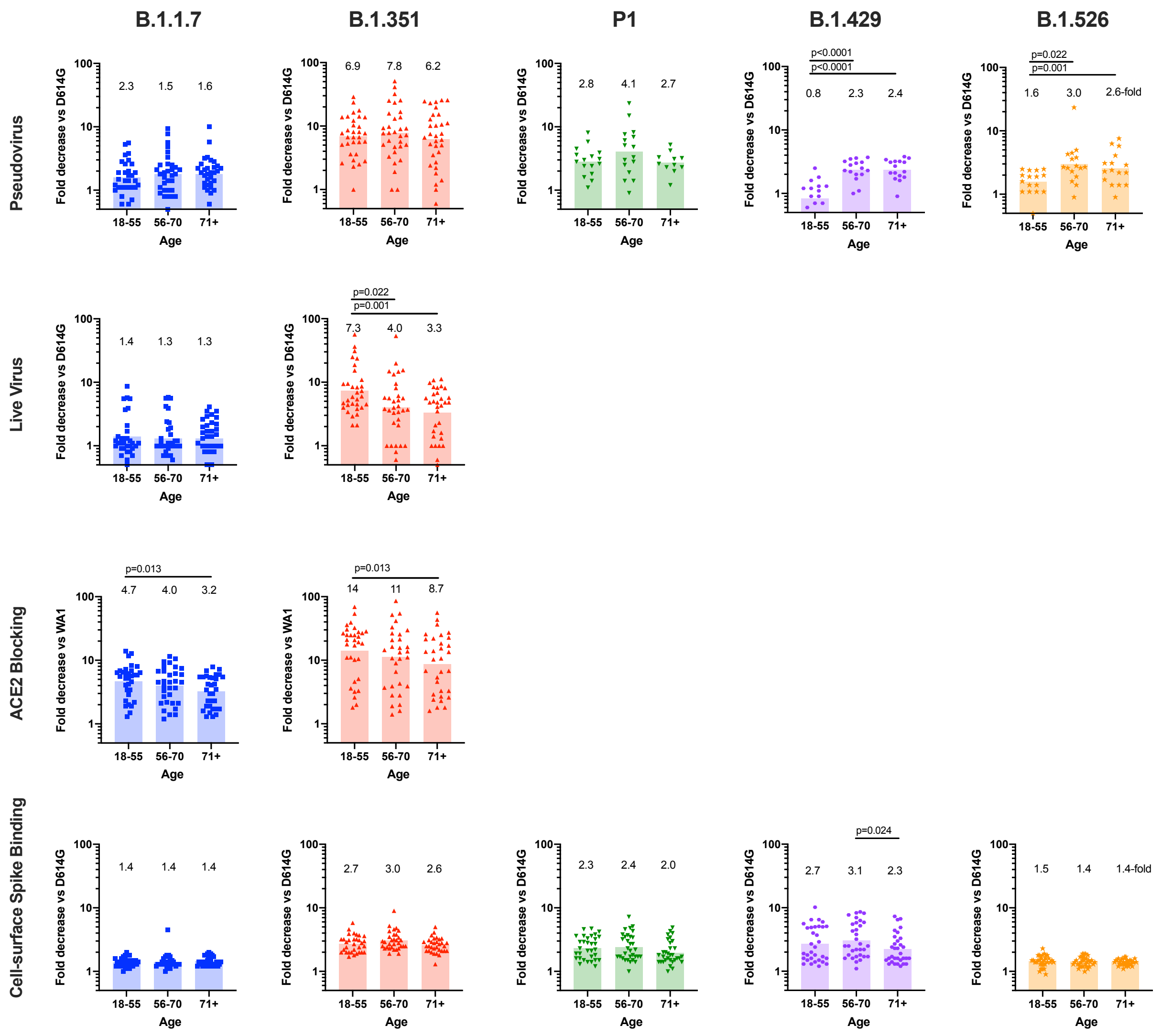

Fig. S3.

Effect of age on relative recognition of variants. Fold reduction in ID50 in each age group (8 subjects, 4 timepoints each, n=32 total) for each variant compared to WA1 or D614G. Bar: geometric mean. p values: Mann-Whitney test; values are not corrected for multiple comparisons; * p=0.01-0.05, ** p=0.001-0.01.

bioRxiv preprint doi: https://doi.org/10.1101/2021.05.13.444010; this version posted May 16, 2021. The copyright holder for this preprint (which was not certified by peer review) is the author/funder. This article is a US Government work. It is not subject to copyright under 17 USC 105 and is also made available for use under a CC0 license.

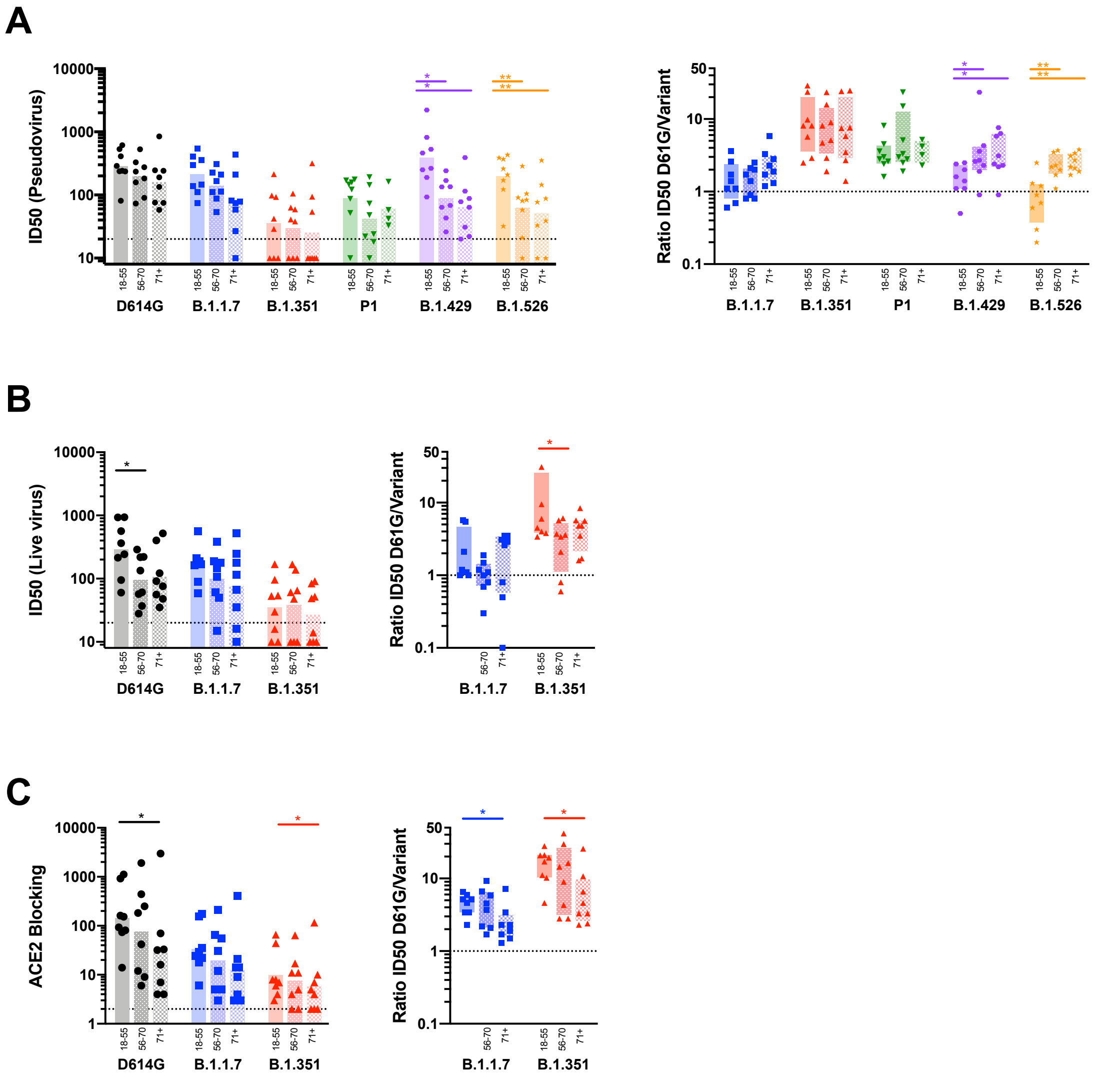

Fig. S4.
Effect of age on relative recognition of variants at Day 209.
Left, assay values for each variant. Bar: geometric mean. Right, ratios compared to WA1 or D614G for each age group (n=8) and variant. p values: Mann-Whitney test; values are not corrected for multiple comparisons; * p=0.01-0.05, ** p=0.001-0.01.
A. Pseudovirus neutralization. **B.** Live-virus FRNT neutralization**.** **C.** ACE2 blocking assay.

bioRxiv preprint doi: https://doi.org/10.1101/2021.05.13.444010; this version posted May 16, 2021. The copyright holder for this preprint (which was not certified by peer review) is the author/funder. This article is a US Government work. It is not subject to copyright under 17 USC 105 and is also made available for use under a CC0 license.

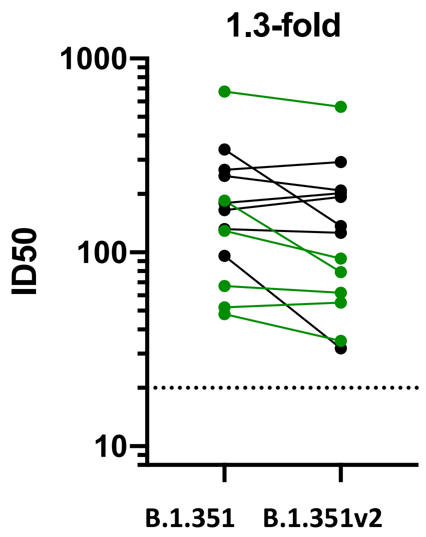

B.1.351	L18F-D80A-D215G-(L242-244)del-R246I-K417N-E484K-N501Y-D614G-A701V
B.1.351v2	L18F-D80A-D215G-(L242-244)del-K417N-E484K-N501Y-D614G-A701V

Fig. S5.
Two versions of B.1.351 yield similar pseudovirus neutralization IC50s. N=13 Sera (black, ages 18-55; green, ages 55-70) were assessed in pseudovirus neutralization assay. The spike proteins in the pseudoviruses differ only at amino acid 246 as indicated.

bioRxiv preprint doi: https://doi.org/10.1101/2021.05.13.444010; this version posted May 16, 2021. The copyright holder for this preprint (which was not certified by peer review) is the author/funder. This article is a US Government work. It is not subject to copyright under 17 USC 105 and is also made available for use under a CC0 license.

**Pseudovirus**	**Name**	**Mutations relative to parental WA1**
	D614G	D614G
	D614G.N439K	N439K-D614G
	D614G.Y453F	Y453F-D614G
	D614G.E484K	E484K-D614G
	D614G.N501Y	N501Y-D614G
	B.1.1.7	d69H/70V-Y144del-N501Y-A570D-D614G-P681H-T716I-S982A-D1118H
	B.1.351	L18F-D80A-D215G-(L242-244)del-R246I-K417N-E484K-N501Y-D614G-A701V
	B.1.351v2	L18F-D80A-D215G-(L242-244)del-K417N-E484K-N501Y-D614G-A701V
	P.1	L18F-T20N-P26S-D138Y-R190S-K417T-E484K-N501Y-D614G-H655Y-T1027I-V1176F
	B.1.429	S13I-W152C-L425R-D614G
	B.1.526	L5F-T95I-D253G-E484K-D614G-A701V
**Cell-surface expressed Spike**	D614G	D614G
	B.1.1.7	d69H/70V-Y144del-N501Y-A570D-D614G-P681H-T716I-S982A-D1118H
	B.1.351	L18F-D80A-D215G-(L242-244)del-R246I-K417N-E484K-N501Y-D614G-A701V
	P.1	L18F-T20N-P26S-D138Y-R190S-K417T-E484K-N501Y-D614G-H655Y-T1027I-V1176F
	B.1.429	S13I-W152C-L425R-D614G
	B.1.526	L5F-T95I-D253G-E484K-D614G-A701V
**S-2P Proteins**	WA1	D614G
	B.1.1.7	d69H/70V-Y144del-N501Y-A570D-D614G-P681H-T716I-S982A-D1118H
	B.1.351	L18F-D80A-D215G-(L242-244)del-R246I-K417N-E484K-N501Y-D614G-A701V
	P.1	L18F-T20N-P26S-D138Y-R190S-K417T-E484K-N501Y-D614G-H655Y-T1027I-V1176F
**RBD Proteins**	WA1	
	B.1.1.7	N501Y
	B.1.351	K417N-E484K-N501Y
	P.1	K417T-E484K-N501Y
**Live Virus**	83E (D614G)[1]	D614G
	B.1.1.7	d69H/70V-Y144del-N501Y-A570D-D614G-P681H-T716I-S982A-D1118H
	B.1.351[2]	L18F-D80A-D215G-(L242-244)-K417N-E484K-N501Y-D614G-A701V

[1]Live virus strain 83E differs from WA1 at a single position in Spike, D614G; there are other differences across the genome, as reported in (Edara et al, 2021 JAMA)
[2]Live virus strain B.1.351 has same spike mutations as B.1.351v2 used for pseudoviruses.

Table S1.

Sequences of spike proteins used in each assay.

bioRxiv preprint doi: https://doi.org/10.1101/2021.05.13.444010; this version posted May 16, 2021. The copyright holder for this preprint (which was not certified by peer review) is the author/funder. This article is a US Government work. It is not subject to copyright under 17 USC 105 and is also made available for use under a CC0 license.

Assay	Variant	Ratio
Pseudovirus Neutralization	B.1.1.7	1.7
	B.1.351	6.9
	P.1	3.2
	B.1.429	1.7
	B.1.526	2.3
Live Virus Neutralization	B.1.1.7	1.3
	B.1.351	4.6
ACE2 Blocking	B.1.1.7	3.9
	B.1.351	11.1
S-2P Binding	B.1.1.7	1.3
	B.1.351	1.9
	P.1	1.7
RBD Binding	B.1.1.7	1.2
	B.1.351	2.7
	P.1	1.9
Cell-Surface Spike Binding	B.1.1.7	1.4
	B.1.351	2.8
	P.1	2.2
	B.1.429	2.6
	B.1.526	1.4

Table S2.

Geometric mean of ratios of value for D614G (pseudovirus, live virus, cell-surface spike binding) or WA1 (ACE2 blocking, S-2P binding, RBD binding) compared to the indicated variant.
